# Successful Dabrafenib Desensitization Protocols in a Patient with Metastatic Melanoma

**DOI:** 10.3390/medicina58040511

**Published:** 2022-04-03

**Authors:** Roxana Silvia Bumbacea, Selda Ali, Sabina Loredana Corcea, Dan Corneliu Jinga, Luiza Spiru

**Affiliations:** 1 Department of Allergology “Carol Davila”, University of Medicine and Pharmacy, 050474 Bucharest, Romania; roxana.bumbacea@umfcd.ro; 2 Department of Allergy and Clinical Immunology, “Dr. Carol Davila” Nephrology Clinical Hospital, 010731 Bucharest, Romania; 3 Department of Pharmacology “Carol Davila”, University of Medicine and Pharmacy, 050474 Bucharest, Romania; sabina.corcea@drd.umfcd.ro; 4 Novomedica Center of Excellence, 030167 Bucharest, Romania; 5 Department of Medical Oncology, Neolife Medical Center, 013812 Bucharest, Romania; danjinga2002@yahoo.com; 6 Department of Geriatrics and Gerontology “Carol Davila”, University of Medicine and Pharmacy, 050474 Bucharest, Romania; lsaslan@brainaging.ro; 7 “Ana Aslan” International Foundation, 020771 Bucharest, Romania

**Keywords:** dabrafenib hypersensitivity, dabrafenib desensitization, metastatic melanoma, personalized treatment

## Abstract

Dabrafenib and trametinib are two available molecules that have been approved for the treatment of metastatic melanoma with BRAF-V600E or V600K mutations. Their combined therapy has led to long-lasting survival benefits and substantially improved outcomes. Until now, only a few cases of severe hypersensitivity reactions to dabrafenib and vemurafenib have been reported, and even fewer desensitization protocols to these molecules have been documented. We report the case of a 71-year-old female patient with metastatic melanoma harboring a BRAF-V600E mutation undergoing targeted therapy with dabrafenib and trametinib. Two weeks after the initiation of the combined treatment, she developed a hypersensitivity reaction. The cause–effect relationship between dabrafenib and the hypersensitivity reaction was demonstrated twice, when symptoms recurred upon dabrafenib reintroduction. We started a rapid 3-day dabrafenib desensitization protocol, which was well tolerated. When the patient discontinued the drug administration, we decided on a longer protocol that included more steps and more days in order to prevent the occurrence of other hypersensitivity reactions. Our patient tolerated both rapid and slow-going schedules, the first one reaching the final dose within 3 days and the second one reaching the total daily dose within 14 days. Depending on the patient’s needs, the severity of the hypersensitivity reaction and the hospital’s availability, the doctor may choose either the rapid or slow-going desensitization protocol.

## 1. Introduction

Dabrafenib, a specific BRAF inhibitor, and trametinib, a MEK inhibitor, are two available molecules approved in Europe and the USA for the treatment of metastatic melanoma with BRAF-V600E or V600K mutations.

Considering the poor prognosis of patients with advanced melanoma, dabrafenib and trametinib combined therapy has led to long-lasting survival benefits and substantially improved outcomes compared with monotherapy [1].

Moreover, the safety profile of this therapy is manageable, and only sparse cases of severe hypersensitivity reactions to BRAF inhibitors (dabrafenib or vemurafenib) have been reported until now [2,3,4,5,6].

However, the clinician might find it challenging to face a severe drug reaction to a mandatory treatment in metastatic melanoma. Therefore, desensitization protocols can help continue the standard therapy, including with the culprit drug. Desensitization is a process that modifies the immunological response to a drug, leading to temporary tolerance, allowing a patient who has suffered from a drug hypersensitivity reaction to safely tolerate the essential medication for an extended period of time [7]. The procedure consists of administering increasing amounts of the drug in a step-by-step manner until the recommended dose has been reached. Once tolerance is achieved, the patient must continue to consume the drug for as long as needed. If treatment is stopped, tolerance is lost and a new protocol should be performed [8,9].

Until now, only two desensitization protocols for dabrafenib have been reported, the first one for an immediate hypersensitivity reaction [5] and the second for a delayed hypersensitivity reaction, toxic epidermal necrolysis [6].

## 2. Case Report

A 71-year-old female patient with a history of metastatic melanoma harboring a BRAF-V600E mutation undergoing targeted therapy with dabrafenib and trametinib was referred to our clinic for an allergy evaluation of a hypersensitivity reaction developed two weeks after the initiation of the above-mentioned treatment. Other comorbidities included arterial hypertension, asthma and gastroesophageal reflux disease, with treatment including bisoprolol 2.5 mg daily, perindopril/indapamide/amlodipine in fixed combination 5/1.25/5 mg daily, salmeterol/fluticasone 50/250 mcg twice daily and pantoprazole 40 mg daily, respectively.

In November 2019, the patient underwent an excision of an ulcerated lesion located on the inner surface of the right calf, which was highly suspicious of melanoma. The histopathological exam confirmed the full excision of the melanoma, with a Breslow index of 5.2 mm, a mitotic index of 11 mitoses/mm2 and free margins.

Subsequently, one month later, a wide local excision and sentinel lymph node biopsy of two inguinal lymph nodes were conducted according to the guidelines [10]. The melanoma was staged as pT4bN2aM0 stage IIIC, BRAF-V600E positive. After receiving 15 regimens of Nivolumab 480 mg, the patient became immuno-resistant to the drug and the disease progressed with satellite and in-transit metastasis requiring excision. In May 2021, the oncologist switched the patient to a combined targeted regimen involving a BRAF inhibitor (dabrafenib 150 mg twice daily) and a MEK inhibitor (trametinib 2 mg once daily). Two weeks after starting the new medication schedule, the patient developed an urticarial rash on the upper and lower limbs associated with systemic symptoms: shivers, malaise, and a cough. The patient was evaluated by the oncologist and the targeted therapy was discontinued, and she was administered oral steroids and H1 and H2 antihistamines. Ten days later, following the resolution of the urticarial rash, the targeted therapy was resumed with dabrafenib as a monotherapy at a reduced dose of 100 mg twice daily. Despite the new regimen, 2 days later, the patient developed another episode of urticaria accompanied by malaise and nausea. This reaction that occurred while the patient was only on BRAF inhibitor therapy demonstrated the relationship between dabrafenib and the hypersensitivity reaction; consequently, the drug was discontinued and the patient received the same urticaria treatment. 

**Figure 1:**
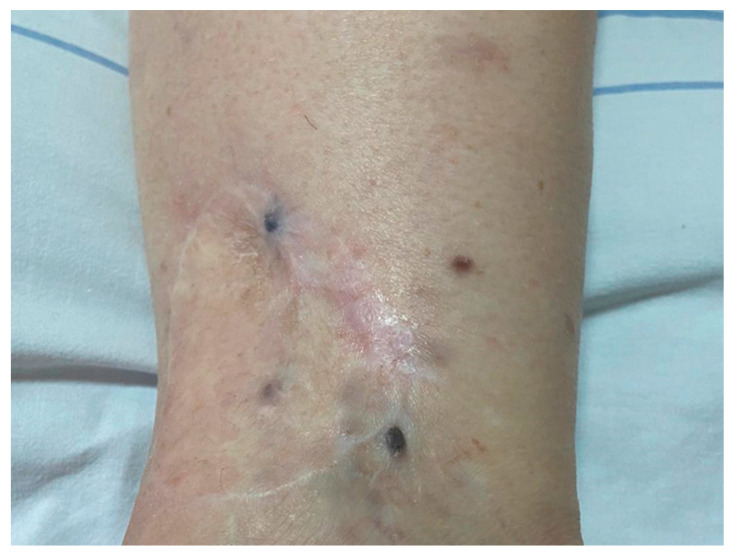
Excised metastatic melanoma on the right ankle with two metastases.

The patient was admitted to the Allergy Department for complete evaluation two weeks after the second hypersensitivity reaction. On clinical examination, the patient presented surgical scars on the right calf, two dark-colored nodules with a diameter of 4 mm each and two dark macules with faded borders in the proximity of the nodules (Figure 1). The total blood count, biochemistry, urinary tests and total IgE were within normal ranges. The spirometry showed normal respiratory function. Skin prick tests to common inhalant allergens were negative. PET-CT showed hypermetabolic masses on the right ankle and at the site of the right inguinal lymph nodes.

An online consulting board involving an oncologist, a dermatologist and an allergist was assembled in order to decide the best treatment option. Due to the lack of other efficient therapeutic alternatives [11], a decision for continuing the combined therapy was made. Therefore, dabrafenib desensitization was the first step, followed by the reintroduction of trametinib.

We decided on the use of an H1 antihistamine (rupatadine 10 mg daily) as premedication 1 h before the desensitization procedure in a rapid regimen. The patient received gradually increasing doses of dabrafenib, as shown in Table 1, reaching the total recommended daily dose in three days. This schedule was adapted after the 3-day aspirin desensitization protocols [12,13] because the final dabrafenib daily dose was approximately the same as the reported aspirin final doses, and also because there is great evidence of the safety and long-time continuity of these protocols [14].

On the fourth day, we reintroduced trametinib at 2 mg in order to achieve the combined therapy as recommended by the oncologist. The patient tolerated the regimen and was discharged, with the strong recommendation of not interrupting the above-mentioned treatment.

One month later, following a change in her antihypertensive treatment according to the cardiologist’s prescription, the patient experienced nausea, vomiting and hypotension, and discontinued all medication. After remission of the symptoms, the patient reintroduced the combined therapy, at lower doses, as recommended by the oncologist, with dabrafenib 75 mg twice daily and trametinib 1 mg daily, but in a couple of days urticaria recurred, similar to the aforementioned episode.

She was re-admitted to our department after being treated for urticaria, and another desensitization schedule was implemented. This time, we decided to use a protocol successfully validated by Bar-Sela et al. [5], but we used the same initial premedication with an H1 antihistamine. We decided to consider systemic corticotherapy, as used in the cited protocol, only if symptoms compatible with a drug-induced hypersensitivity reaction occurred. We had to slightly modify the regimen of doses (50 mg × 2, 75 mg, 100 mg instead of 50 mg × 3, 150 mg) on day 14 due to medication side effects: nausea and cough (Table 2). The symptoms improved after adding famotidine and domperidone to the H1 antihistamine premedication.

H1 antihistamine premedication was administered every day, an hour before the desensitization procedure. Except on day one when only one dose was given, all other dabrafenib doses were administered at 30 min intervals. The doses were prepared using the analytical balance in the hospital’s pharmacy. The patient tolerated the total daily dose of dabrafenib. On the last day, we reached the total dose of 300 mg dabrafenib and trametinib 2 mg was added.

At the time of writing the present article, the patient was on the above-mentioned medication regimen, with no reported side effects. She achieved a partial response to dabrafenib/trametinib therapy, defined as more than 50% remission of the target lesions. Given the obvious clinical response, it seems that the desensitization procedure and the interruptions due to hypersensitivity reactions, as well as the dose change imposed by toxicity, did not affect the patient’s prognosis. Otherwise, the temporary cessation and resumption of treatment were performed in accordance with the manufacturer’s recommendations.

## 3. Discussion

Although dabrafenib-induced cutaneous reactions, such as maculopapular rash, are frequently reported [15,16], severe systemic reactions, such as anaphylaxis, have rarely been described [3,5]. Two weeks after the initiation of dabrafenib and trametinib combination therapy, our patient reported a systemic reaction with urticaria, shivers, malaise, and cough. The causal relationship between dabrafenib intake and the hypersensitivity reaction was proven by introducing solely dabrafenib and observing the recurrence of symptoms. There are no published data regarding the diagnostic value of lab tests [17], nor of skin (prick and intradermic) tests; the possible irritant effect of dabrafenib on the skin is unknown. Tryptase levels have not been measured during the reactions. However, the patient reacted on two occasions after the reintroduction of dabrafenib following the hypersensitivity reaction.

The tolerance achieved through drug desensitization protocols usually lasts for as long as the pharmacological therapy is administered without interruption [8]. If drug intake is discontinued, a new desensitization protocol should be started. In our case, the first protocol was adapted from the published aspirin desensitization protocols [12,13]. When selecting a desensitization strategy, the intensity and type of the initial hypersensitivity reaction(s), the patient’s comorbidities and the urgency of intervention should all be taken into account. We opted for a rapid protocol in order to shorten the period of time spent by the patient in hospital during the COVID-19 pandemic and to reach the recommended dose of the vitally important medication as early as possible. On the second hospitalization in the context of a new hypersensitivity reaction and of the patient’s reactive anxiety, we decided on a longer protocol that included more steps and more days in order to slowly increase the daily dose and prevent the occurrence of other hypersensitivity reactions. She tolerated both schedules; the first, the rapid one, reaching the final dose within 3 days, and the second one reaching the total dose within 14 days. Depending on the patient’s needs, the severity of the hypersensitivity reaction and the hospital’s availability, the doctor may choose either one. The desensitization protocol should take into consideration the balance between the reduction of other side effects of the medication and the urgency of reaching the recommended dose. Continued vigilance and monitoring are essential for a successful, uninterrupted treatment. 

Regarding the 14 days protocol, we did not use corticotherapy in the premedication scheme, as performed by the authors that published the first successful desensitization [5]. Our patient received daily premedication with an H1 antihistamine an hour before dabrafenib was started. When gastrointestinal side effects occurred, an H2 antihistamine and prokinetics were added. The patient tolerated the recommended dose with minimum side effects.

## 4. Conclusions

As dabrafenib and trametinib combination therapy is frequently prescribed as part of elective therapy in metastatic melanoma with BRAF V600E or V600k mutations, hypersensitivity reactions may occur. In patients where treatment is considered to be vital and with no other equally efficient therapeutic options available, desensitization to the culprit drug may be a life-saving procedure. We report a dabrafenib hypersensitivity reaction to which two desensitization protocols were successfully applied: a rapid 3-day desensitization schedule and a slow-going 14-day protocol.

## Figures and Tables

**Table 1:** Protocol 1 of dabrafenib desensitization.

	1st Dose (mg)	2nd Dose (mg)	3rd Dose (mg)	4th Dose (mg)	5th Dose (mg)	6th Dose (mg)	7th Dose (mg)	Total Daily Dose (mg)
Day 1 *	5	10	15	25	45	50	-	150
Day 2 **	50	50	100	-	-	-	150	200
Day 3 ***	150	-	-	-	-	-	150	300

* On the first day, the patient received 7 doses, each at 1-hour interval. ** On the second day, the first 3 doses were administered at 1-hour interval, the fourth dose was administered 12 h after the first dose. *** On the third day, 2 doses at a 12 hours interval were given.

**Table 2:** Protocol 2 of dabrafenib desensitization (adapted after Bar Sela et al. [5]).

	1st Dose (mg)	2nd Dose * (mg)	3rd Dose * (mg)	4th Dose * (mg)	Total Daily Dose (mg)
Day 1	15	-	-	-	15
Day 2	15	15	-	-	30
Day 3	15	15	15	-	45
Day 4	15	15	15	15	60
Day 5	15	15	15	30	75
Day 6	15	15	30	30	90
Day 7	15	30	30	30	105
Day 8	30	30	30	30	120
Day 9	30	30	30	50	140
Day 10	30	50	50	50	180
Day 11	50	50	50	50	200
Day 12	50	50	50	75	225
Day 13	50	50	75	75	250
Day 14	50	50	75	100	300

* The time interval between the doses was 30 min.

## Data Availability

The data presented in this study are available on request from the corresponding author.
